# Uncovering the different scales in deer–forest interactions

**DOI:** 10.1002/ece3.7439

**Published:** 2021-03-24

**Authors:** Juan Ignacio Ramirez

**Affiliations:** ^1^ Department of Ecology and Environmental Sciences Umeå University Umeå Sweden; ^2^ Environmental Science Group University & Research Wageningen the Netherlands; ^3^ Colegio de Ciencias Biológicas y Ambientales COCIBA Universidad San Francisco de Quito USFQ Quito Ecuador

**Keywords:** boreal, browsing, density, hierarchical, landscape ecology, plant–herbivore interactions, temperate, temporal, spatial

## Abstract

Deer are regarded to be a keystone species as they play a crucial role in the way an ecosystem functions. Most deer–forest interaction studies apply a single scale — process of analyzing ecological interactions by only taking into account one dependent variable — to understand how deer browsing behavior shapes different forest components, but they overlook the fact that forests respond to multiple scales simultaneously. This research evaluates the effect of browsing by wild deer on temperate and boreal forests at different scales by synthesizing seminal papers, specifically (a) what are the effects of deer population density in forest regeneration? (b) What are the effects of deer when forests present diverging spatial characteristics? (c) What are the effects on vegetation at different temporal scales? and (d) What are the hierarchical effects of deer when considering other trophic levels? Additionally, a framework based on modern technology is proposed to answer the multiscale research questions previously identified. When analyzing deer–forest interactions at different scales, the strongest relationships occur at the extremes. For example: when deer assemblage occurs in low or high density and is composed of a mix of small and large species. As forests on poor soils remain restrained in size, isolated and chronically browsed. When forests harbor incomplete trophic levels, the effects spill over to lower trophic levels. To better understand the complexities in deer–forest interactions, researchers should combine technology‐based instruments like fixed sensors and drones with field‐tested methods such observational studies and experiments to tackle multiscale research questions.

## Graphical abstract

When analyzing deer–forest interactions at different scales, the strongest relationships occur at the extremes: when deer assemblage occurs in low or high density and is composed of a mix of small and large species. As forests on poor soils remain restrained in size, isolated and chronically browsed. When forests harbor incomplete trophic levels, the effects spill over to lower trophic levels. At this point, deer effects in forest might be irreversible. To overcome the challenge of understanding how vegetation responds to deer browsing and trampling at multiple scales, combining modern technology with field‐tested methods could provide a holistic insight into deer–forest interactions.

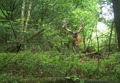

## 1 INTRODUCTION

**FIGURE 1:**
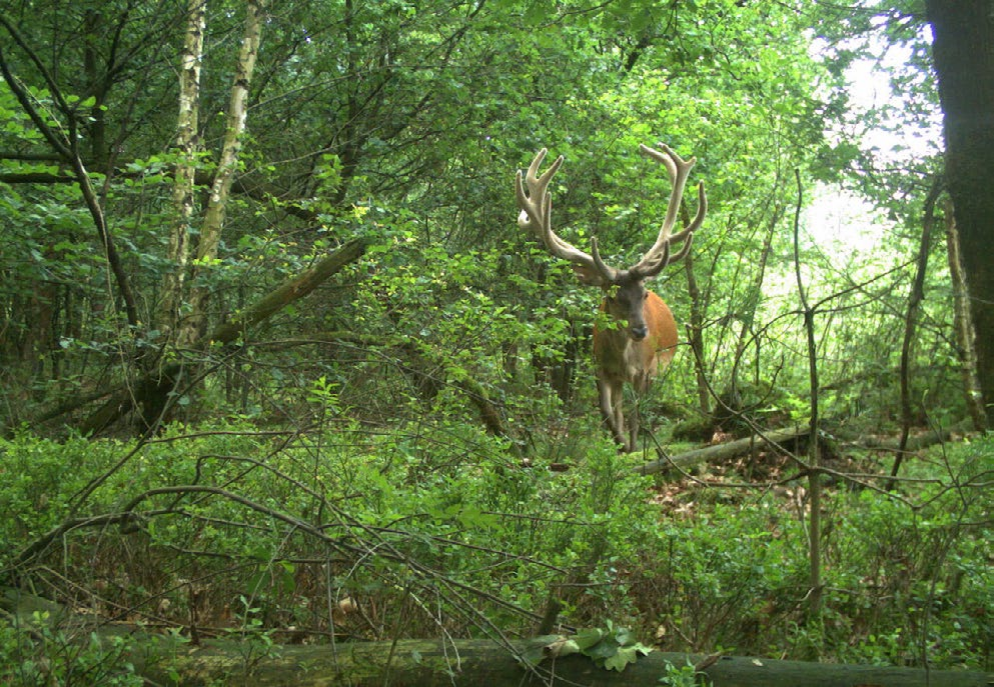
Deer interact with forests at different scales. In this picture, a red deer (*Cervus elaphus*) roaming around the forest. Picture taken in the Veluwe, the Netherlands

The animals that belong to the Cervidae family are commonly known as deer, and some of the most important members from the northern hemisphere are *Cervus canadensis* (wapiti), *Cervus elaphus* (red deer), *Dama dama* (fallow deer), *Rangifer tarandus* (reindeer), *Capreolus capreolus* (roe deer), *Odocoileus hemionus* (mule deer), *Odocoileus virginianus* (white‐tailed deer), and *Alces alces* (moose). Deer interact with forest directly by browsing, trampling, fraying, and stripping vegetation and indirectly by seed dispersing and defecating (Ramirez et al., 2018, 2019). Across different biomes, deer are regarded to be a keystone species for the following reasons: (a) These animals have a disproportional effect on the vegetation community relative to their abundance, (b) they play a crucial role in the way an ecosystem functions like forest succession and nutrient cycle (Waller & Alverson, 1997), and (c) their feeding behavior can lead to shifts in forest species composition (Figure 1) (Coverdale et al., 2016; Mathisen et al., 2010).

In the past decades, deer (referring to populations of wild deer) in the northern hemisphere have increased in abundance (Reimoser, 2003) due to rewilding programs, abandonment of agricultural land, competitive release from domestic ungulates, absence of top predators, stricter hunting regulations, and improvement of habitat quality (Kuiters et al., 1996; Rooney, 2001). With an increasing population density, deer may lead to an excessive top‐down control on forest regeneration, which may accumulate in time and trigger cascading effects on lower trophic levels (Ramirez et al., 2020).

Most studies on deer–forest interactions apply a one‐ or a two‐scale approach to disentangle the mechanism of how deer shape forest structure, composition, and succession, when in fact, forests respond to multiple scales at once (Kuijper et al., 2013; Nuttle et al., 2014; Persson et al., 2005). Hence, this review evaluates the effects of wild deer populations on temperate and boreal forests at different scales, specifically (a) the effect of deer population density in forest regeneration, (b) the effects in forest with diverging spatial characteristics, (c) the effect on forest vegetation at different temporal scales, and (d) the hierarchical effects when including other trophic levels. Finally, a framework based on modern technology is proposed to answer the multiscale points previously identified.

## 2 DEER DENSITY SCALE

Deer density is shaped by top‐down control (predation and culling) and bottom‐up control mechanisms (food quality and availability). Top‐down control by predation and culling directly reduces deer density (McGraw & Furedi, 2005). Bottom‐up control by plants can also decrease deer density by limiting food availability. Deer are controlled by a combination of top‐down and bottom‐up mechanisms and yet, deer density is also shaped by other factors. Body mass determines deer density because large animals have large per capita food requirements and occur therefore at lower densities than small animals (Damuth, 1987). Reproductive behavior of “r” and “k” strategy animals defines offspring numbers and thus probable species local density (Pianka, 1970). Competition also influences animal density because at high population densities, deer scare away competition (Courchamp et al., 1999) but are safer from predators (Brown et al., 1999; Hager & Helfman, 1991).

Deer density might be the most important scale in shaping temperate and boreal forests. In general, a high deer density leads to a shift in canopy composition by browsing palatable species in the understory and thus allowing only conifers and a few broadleaves reaching the forest canopy (Ramirez et al., 2019). At medium density, in accordance with the Intermediate Disturbance Hypothesis (Wilkinson, 1999), deer favor ferns and sometimes yellow birch by browsing and creating open spaces in the understory for species to establish and develop (Rooney & Waller, 2003). At low density, forests will have low plant diversity because open spaces in the understory are not created, so light‐demanding species cannot establish and develop (Rooney & Waller, 2003).

In a global effort to unveil the relationship between temperate and boreal forests with deer population size, a meta‐analysis traced a curvilinear dose–response relationship between ungulate density (mainly deer) and forest composition (Ramirez et al., 2018). This same curvilinear response was confirmed in a field study that paired camera traps to vegetation plots in a mixed temperate forest (Ramirez et al., 2021). Furthermore, the meta‐analysis study identified tipping points in forests when the effect of ungulate density switched from neutral to negative. In 70% of the evaluated cases, ungulate density had a negative effect. Critical tipping points, where ungulate started to have a negative effect on forest regeneration, were found at an ungulate metabolic weight density of 115 kg/km^2^ for forest regeneration, 141 kg/km^2^ for forest structure, and 251 kg/km^2^ for forest functioning, which is roughly equivalent to 10, 13, and 23 roe deer per km^2^. These results propose that, regardless of the unique spatial characteristic of each location, a high ungulate density tends to reduce sapling diversity and density and these effects may build up over time.

A higher deer density combined with trampling directly damages vegetation tissue or indirectly limits vegetation growth by compacting the upper soil layers (Pellerin et al., 2006). By doing so, it limits water retention, soil aeration, and nutrient cycling (Hättenschwiler et al., 2005; Lavelle et al., 1992). A higher deer density evidently increases soil compaction; however, trampling is also dependent on the composition of the deer assemblage, with larger deer having stronger effects (Duncan & Holdaway, 1989).

Deer effects on forest are also mediated by the composition of the deer assemblage and the manner they select the browsing patches. For example, small deer are forced to feed more selectively compared to larger deer because of their small size gut (Bunnell & Gillingham, 1985). When small and large deer live in the same forest, small deer tend to select poorer forest patches in accordance with the Optimal Foraging Theory (MacArthur & Pianka, 1966). The ecological characteristics of the species also determine browsing selection and intensity; for example, strict browser — such as roe deer and moose—can browse intensively on palatable and less palatable trees because they are forest species, whereas intermediate browsers—such as red and fallow deer—spend part of their time between grazing lawns and forest patches (Gill, 1992), suggesting that the spatial arrangement of forests also plays a role on forest structure and composition.

## 3 FOREST SPATIAL SCALE

Studies from countries across temperate and boreal regions — including Poland (Kuijper et al., 2010), the Netherlands (Ramirez et al., 2019), Sweden (Mathisen et al., 2010), United States (Asnani et al., 2006), Canada (Allombert et al., 2005), Argentina (Barrios‐Garcia et al., 2012), Japan (Suzuki et al., 2013), and New Zealand (Husheer et al., 2003) — found that vegetation responses to deer are highly heterogeneous. Most studies presented decreasing relationships (Holm et al., 2013; Nuttle et al., 2014), with notable exceptions (Eycott et al., 2007; Royo et al., 2010). The underlying reason for this wide variation in results — besides deer density — is thought to be related to spatial characteristics of each study location, such as primary productivity, soil fertility, and forest size.

Primary productivity in combination with soil fertility allows plants to better cope with herbivory as resources are not limited. For example, seedlings, under constant supply of light, water, and nutrients, can grow fast to escape the browsing height, which typically is below 220 cm (Walters et al., 2020). They are able to develop side shoots to physically protect the apex shoot from herbivory (Gill & Beardall, 2001), allocate energy for reproduction to ensure positive demographics as flowers are severely affected by herbivory (Lehtilä & Strauss, 1999; Rooney & Gross, 2003), and develop chemical and physical defenses to reduce palatability (Lindroth & St. Clair, 2013).

Forest size also brings limitations in terms of their ability to support herbivory. In general, small forests have stronger edge effect due to perimeter–area relationships causing skewed patterns of use in wild animals, which rises several issues (Murcia, 1995). Deer and predators tend to avoid edge areas because they are highly degraded by human interference, concentrating herbivory in the forest interior (Cadenasso & Pickett, 2000). Edge areas across temperate and boreal systems are rapidly colonized by competitive plant species which in time can spread to the interior of the forest (Sumners & Archibold, 2007; Yates et al., 2004). By then, the entire stability of the system may be compromised because of potential changes in food supply for animals and nutrient cycle in soil (Murcia, 1995). Also, small forests tend to be isolated from other forests and are more likely to experience higher rate of fragmentation. Hence, large animal species cannot migrate and are subjected to stochastic extinction (Woodroffe & Ginsberg, 1998). These spatial effects that govern forest are not entirely understood, and neither is the role they play in the long term because fragmentation, soil formation, and forest succession may exceed human lifespan.

## 4 FOREST TEMPORAL SCALE

Temperate and boreal forest succession is characterized by having light‐demanding tree species in early‐successional stages and shade‐tolerant species in late‐successional stages (Ramirez et al., 2019). These shifts in tree species composition make forest more or less susceptible to browsing effects. Early‐successional stages in general are prone to greater browsing effects than late successional because at this stage, plants are within the browsing height due to their small size (Walters et al., 2020). Herbivory keeps trees in small size classes and prompts stronger competition for resources between trees, shrubs, and herbs (Gill & Beardall, 2001). Similarly, plant composition in early‐successional stages of a mixed temperate forest is dominated by palatable tree species that lack chemical and physical defenses against herbivory (i.e., broadleaves), whereas in late‐successional stages, trees are armed with chemical and physical defenses (i.e., bark antifeedants in conifers), which makes them less prone to herbivory (Kuiters & Slim, 2002).

To provide a better understanding on how forest succession unfolds in a situation of chronic browsing and trampling, several studies have evaluated short‐ and long‐term impacts of deer populations in temperate and boreal forests. In the short term, deer browsing halts plant size and density in unfenced plots compared to fenced plots (Ramirez et al., 2019), yet vegetation composition remains unchanged (Gordon & Prins, 2008), possibly because herbivory damage needs to accumulate over several growing seasons before exhibiting shifts in vegetation composition. In the long term, chronosequence studies comparing paired fenced and unfenced plots — ranging in age from 1 to 33 years since establishment — presented a significant difference in forest composition, structure, and succession (Ramirez et al., 2019). Fenced plots, where deer were excluded, experienced higher canopy cover, tree species richness, and a thicker litter layer. Fenced plots were also associated with late‐successional tree species, while unfenced plots were associated with early‐successional species. This indicates that deer halts natural succession by keeping the forest in an early‐successional stage. These results highlight an important mismatch between the short‐ and the long‐term scale effects of deer in forests. At this point, it remains unclear whether these long‐term effects can trigger cascading effects on lower forest trophic levels.

## 5 DEER HIERARCHICAL SCALE

A great number of studies have looked into the strong top‐down control exerted by deer on vegetation, but fewer studies have investigated if these effects spill over to other forest trophic levels. Empirical evidence indicates that, by changing vegetation composition and structure, deer browsing can have impacts on the species diversity of invertebrates, rodents, and birds (Allombert, Gaston, et al., 2005; Allombert et al., 2005; Buesching et al., 2011). A temperate forest study traced the cascading effects that deer have on a semi‐complete forest community, including vegetation, soil invertebrates and rodents, and a set of ecosystem properties and functions, such as soil quality, litter decomposition, and nutrient mineralization (Ramirez et al., 2020). This was done by surveying different trophic levels in a network of fenced and unfenced plots and estimating deer abundance outside the fenced plots with camera traps (Ramirez et al., 2020). Specifically, deer presence decreased sapling density presumably by browsing and trampling, which indirectly decreased rodent activity because rodents are more exposed to climatic events and predation (Flowerdew & Ellwood, 2001). Deer presence decreases sapling density by browsing and trampling, which indirectly decreases rodent activity due to overexposure to climatic events and predation. Deer trampling decreases litter depth by mixing soil with litter (Hobbs, 1996), which indirectly reduces macroinvertebrate diversity due to litter being an important microhabitat for food and shelter, as well as a protective layer that controls soil humidity, temperature, and light (Mills & Macdonald, 2004). Deer trampling also increases soil compaction, which in turn decreases invertebrate diversity because high soil compaction limits soil water storage, soil aeration, and invertebrate movement (Althoff & Thien, 2005; Lal, 1988). Litter decomposition and nutrient mineralization were found not to be influenced by deer presence. These cascading effects were even stronger when linking different trophic levels and forest components to deer abundance, revealing the important role of deer in this forest (Ramirez et al., 2020).

With the extirpation of top predators from temperate and boreal forests, the hierarchical power that deer have over lower trophic level is reinforced as deer populations are not controlled by predation. In other words, predation absence allows deer effects to spill over to lower trophic levels and reinforces the density dependent effects. This was observed in a Polish study where deer effects on vegetation were less strong in a wolf core area, compared to the periphery (Kuijper et al., 2013). Future research should continue to include additional trophic levels than the ones described here, as it is suspected that herbivory and anthropogenic effects influence many more trophic levels than what it is traditionally believed, including reptiles, amphibians, small predators, and scavengers.

## 6 INTERLINKS BETWEEN SCALES

**FIGURE 2:**
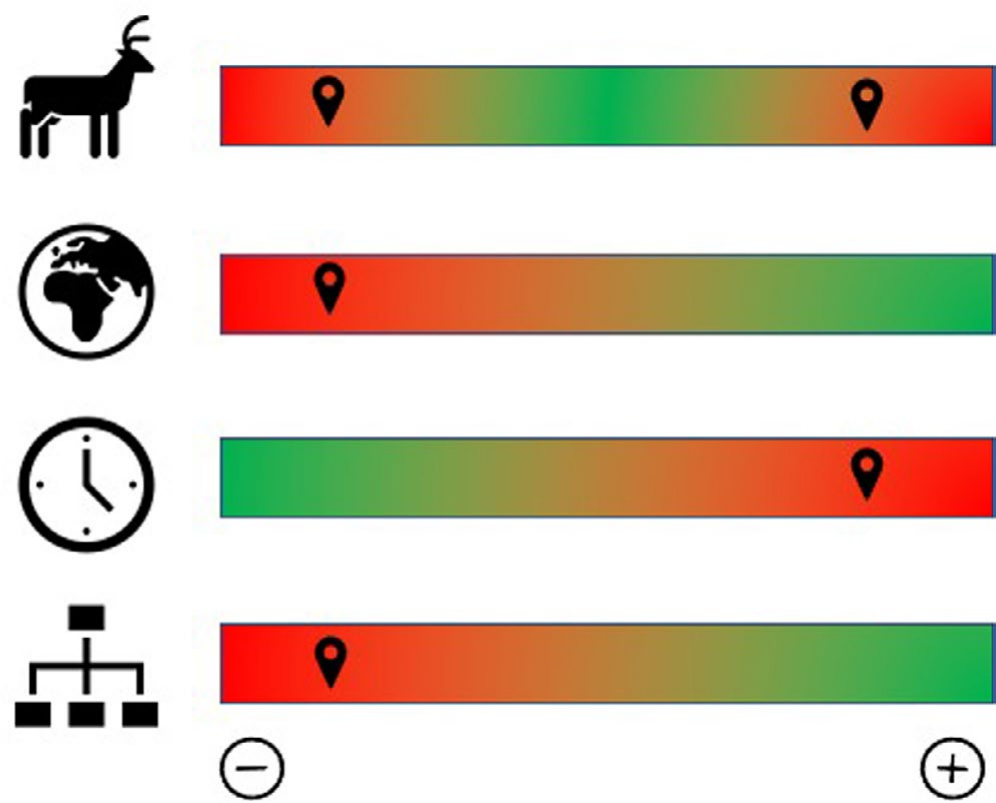
Deer effects at a multiscale approach. Deer effects are stronger (red scale color and location icon) on forests when deer assemblage occurs in low or high density and is composed of a mix of small and large species (deer icon). As forests on poor soils remain restrained in size and isolated (planet icon), while forest are subject to chronic browsing since early‐succession stage (watch icon). When forests harbor incomplete trophic levels, the effects spill over to lower trophic levels (organogram icon)

The relationships between deer and forests drastically change according to the characteristics of the scales: deer density, forest spatiality, temporal succession, and extent of hierarchy (Cromsigt & Kuijper, 2011; Liang & Seagle, 2002; Ramirez et al., 2018, 2019). Thus, certain effects of herbivory and trampling are easy to predict, while others are very complex. It seems evident that deer are keystone species and their relationships with forests are characterized by being nonlinear (Ramirez et al., 2021). This review has shown so far that it is likely that deer effects on vegetation are stronger at the extremes of each of the scales discussed (Figure 2): (a) when deer assemblage occurs at low or high density and is composed of a mix of small and large species; (b) As forests on poor soils remain restrained in size and do not possess wildlife corridors so deer can migrate from one foraging ground to another; (c) while forests are subject to chronic browsing from early‐successional stage, and (d) when forests harbor incomplete trophic chains, the effects are much stronger in vegetation and spill over to lower trophic levels (i.e., rodents and invertebrates) and ecosystem processes (organic matter decomposition and nutrient mineralization).

## 7 A HOLISTIC APPROACH TO STUDY DEER–FOREST INTERACTIONS

The interactions between deer populations and ecosystems are difficult to disentangle because they form part of complex systems (Weisberg & Bugmann, 2003). To truly understand the role of deer in temperate and boreal forests, it is necessary to develop a more holistic framework relying on technology that is capable of answering multiscale research questions. This framework integrates questions that incorporate two different approaches: scaling‐down and scaling‐up. A scaling‐down approach aims to understand the mechanisms involved in the interactions between deer and forests, which can be done both by recreating these interactions in a laboratory or greenhouse and by conducting field experiments. In turn, a scaling‐up approach evaluates the interactions in the context of the system, for example, in forest at different successional stages, differing in primary productivity and size, variation in hunting and predation regimes, and different levels of human interference. This can be done by conducting extensive and longitudinal studies in the field complemented with modeling techniques. More importantly, proven technologies can help to bridge the gap between researching deer–forest interactions and understanding those same interactions in the context of the system.

### 7.1 Scaling‐down approach

Confounding factors (e.g., light availability, soil fertility, distance to nearest road) can provide a competitive advantage to either plants or herbivores, thus having the potential to entirely shift deer–forest interactions. To contextualize their effect, experiments can be conducted in controlled environments that isolate deer mechanism from the confounding factors that are present in forests (Putman, 1996; Weisberg & Bugmann, 2003). For example, (a) the mediating effect of primary productivity on deer–forest interactions can reveal the plant defense mechanisms to browsing as with higher nutrient availability, plants can allocate more energy for fighting herbivory (Lindroth & St. Clair, 2013); (b) predation risk and human presence can directly or indirectly shape the foraging behavior in deer taking effect at both the temporal and spatial scales. That is, deer increase vigilance over browsing time in high‐risk predation areas, and deer select closed forest patches instead of open ones to reduce the likelihood of being spotted by predators (Brown, 1988; Brown et al., 1999; Tufto et al., 1996); (c) deer impacts on forests can change in response to the composition of the deer assemblages and the way they select the browsing patches (Gill, 1992); and (d) the successional stage of a forest may influence deer impacts on vegetation via changes in canopy cover, determining which forest patches are for resting and which ones are for foraging (Putman, 1996; Reimoser & Gossow, 1996). Providing critical points and thresholds to indicate when deer impacts on vegetation are stronger for each confounding factor can simplify our understanding of how complex systems behave, as well as providing guidelines for forest management (Reimoser et al., 1999).

### 7.2 Scaling‐up approach

Deer–forest interactions should also be studied with a scaling‐up approach, especially at spatial and temporal scales (Hobbs, 1996; Weisberg & Bugmann, 2003). By upscaling and including confounding factors, it is possible to better understand how forest systems work. For example, (a) deer at a landscape scale might increase overall plant diversity by creating opportunity for rare species; thus, incorporating a typical sample size for alpha diversity studies will not capture the entire diversity. Future research should choose larger study areas and increase sample size to accurately assess alpha, beta, and gamma diversity (Chollet et al., 2013); (b) because deer home range (e.g., 4.5–10.4 km^2^ for red deer) typically exceeds the study area for vegetation surveys, it is necessary to survey vegetation at a landscape level to better understand deer–forest interactions (Gill, 1992; Gill & Morgan, 2010); (c) small fenced plots are commonly used to study animal effects on vegetation because of their lower labor costs compared to large fenced plots. Therefore, fenced plots are typically associated with spatial scale effects and thus the relationship between deer and vegetation might also be misinterpreted (Wiens, 1989). This can be solved by increasing the size of fenced plots or by using natural islands reflecting deer presence–absence (Allombert, Stockton, et al., 2005); and (d) deer and vegetation surveys used to determine browsing effects in vegetation and habitat use usually incorporate temporal scale effects due to surveys being conducted at one moment of time. By tagging individual trees and following them through time, the temporal scale effect problem can be avoided, providing a better understanding of the interactions between deer and forest successional stages (Schneider, 2001).

As previously stated, scaling‐up and scaling‐down approaches are highly recommended for ecological research in the 21st century because by downscaling, the mechanisms unveil, while upscaling tests whether these mechanisms are still ecologically relevant in the field. Combining a multiscale approach demands a substantial increase in human effort and capital investment and thus, this approach can be challenging to implement in practice. New and affordable technology may thus allow to overcome these scaling challenges by reducing the time needed to collect reliable and systematic information.

### 7.3 Overcoming challenges with technology

The largest obstacle within this field of research is acquiring systematic and replicable information that accurately represents deer–forest interactions (Gill, 1992; Putman et al., 2011). Technology could provide a way forward in different ways. For example, small size sensors detect stress in trees by quantifying changes in sap flow and growth; phenocams can be used to evaluate plant composition and growth throughout the year; terrestrial LiDAR can quantify biomass and understory structure; camera and audio traps can be used to determine deer assemblage composition, evaluate foraging behavior, and quantify hunting intensity; GPS trackers and heart rate monitors can be used to track animal movement and activity; and drones can be used to survey landscape vegetation cover and track animal movement in open areas. However, most scientific advances will undoubtedly occur by combining these technologies.

The Internet of Things—objects that are embedded with all sorts of monitors that are connected by wireless networks with the purpose of exchanging information in a near real time—might be the platform that links the previously described sensors while transmitting the information to a data processing center. At this stage, the greatest bottleneck is processing large amounts of data in short periods of time, and Machine Learning is the option forward. There are numerous applications where The Internet of Things has been used to better understand ecological systems, including the underground behavior of rodents and movement of birds, and also to monitor the Qinling Mountain reserve and help preserve its endemic species (Guo et al., 2015).

## 8 CONCLUSION

The best way to tackle the multiscale dependency in deer–plant interactions is by mounting flexible and reliable technological networks that provide replicable data and with enough resolution. This can be achieved in temperate and boreal systems by developing a long‐term wireless network (i.e., temporal scale) embedded in a large and heterogeneous experimental forests (i.e., spatial scale) and mounting sensors that track ecosystem properties, but also specimens belonging to different trophic levels (i.e., density and hierarchical scales). By doing so, researchers can quantify the interactions among members of the forest community but also, with their environment, even when the system is characterized for being cryptic and highly dynamic.

## CONFLICT OF INTEREST

No conflict of interest to declare.

## AUTHOR CONTRIBUTION

**Juan Ignacio Ramirez:** Conceptualization (lead); Formal analysis (lead); Funding acquisition (lead); Investigation (lead); Project administration (lead); Validation (lead); Visualization (lead); Writing‐original draft (lead); Writing‐review & editing (lead).

## Data Availability

There are no data associated with this publication.
